# Supplementary material for: The interactome of intact mitochondria by cross-linking mass spectrometry provides evidence for coexisting respiratory supercomplexes
Source: Mol Cell Proteomics. 2017 Dec 8;17(2):216–32. doi: 10.1074/mcp.RA117.000470 (PMC5795388; doi:10.1074/mcp.RA117.000470)
Supplement: Supplemental Data [file supp_RA117.000470_133922_0_supp_23978_fzffwf.zip › spectra_annotation/new_mito_protein_spectra_annotation/380-1-5-1-2254-1.pdf]

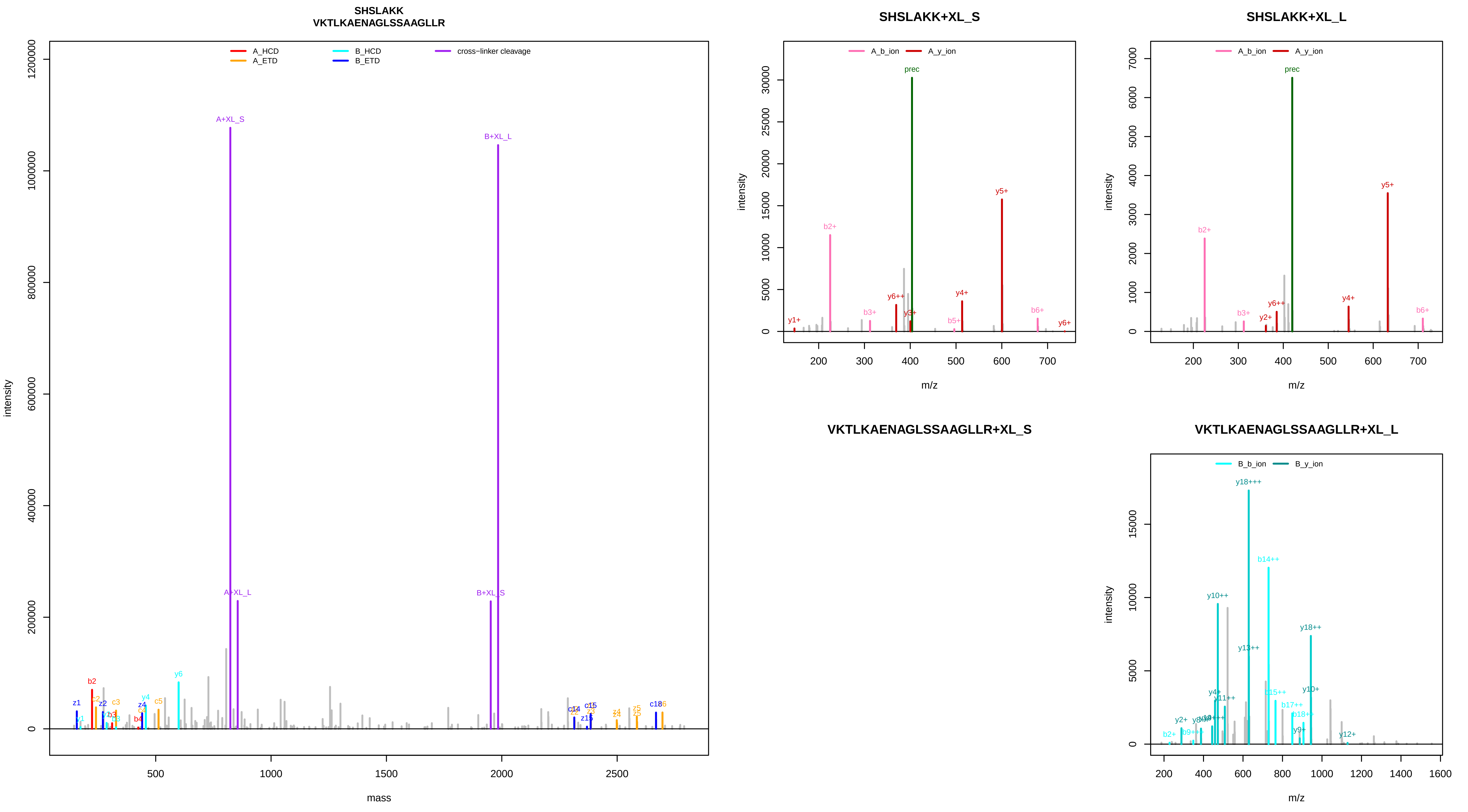
SHSLAKK
VKTLKAENAGLSSAAGLLR
A_HCD
A_ETD
B_HCD
B_ETD
cross−linker cleavage
A+XL_S
B+XL_L
A+XL_L
B+XL_S
intensity
mass
0
200000
400000
600000
800000
1000000
1200000
500
1000
1500
2000
2500
b2
y6
c15
c18
SHSLAKK+XL_S
A_b_ion
A_y_ion
prec
y5+
b2+
y6++
y4+
b3+
b6+
y1+
b5+
y6+
m/z
SHSLAKK+XL_L
y2+
y6++
VKTLKAENAGLSSAAGLLR+XL_S
VKTLKAENAGLSSAAGLLR+XL_L
B_b_ion
B_y_ion
y18+++
b14++
y10++
y18++
y13++
y10+
b15++
b17++
y4+
y11++
y2+
b2+
b9+
y9+
y12+
